# The role of CD8^+^ regulatory T cells and B cell subsets in patients with COVID-19

**DOI:** 10.55730/1300-0144.5388

**Published:** 2022-06-18

**Authors:** Hülya UÇARYILMAZ, Dilek ERGÜN, Hüsamettin VATANSEV, Hande KÖKSAL, Onur URAL, Uğur ARSLAN, Hasibe ARTAÇ

**Affiliations:** 1 Department of Medical Biology, Faculty of Medicine, Selçuk University, Konya, Turkey; 2 Department of Chest Disease, Faculty of Medicine, Selçuk University, Konya, Turkey; 3 Department of Medical Biochemistry, Faculty of Medicine, Selçuk University, Konya, Turkey; 4 Department of General Surgery, Hamidiye Faculty of Medicine, University of Health Sciences, Konya, Turkey; 5 Department of Infectious Diseases and Clinical Microbiology, Faculty of Medicine, Selçuk University, Konya, Turkey; 6 Department of Medical Microbiology, Faculty of Medicine, Selçuk University, Konya, Turkey; 7 Department of Pediatric Immunology and Allergy, Faculty of Medicine, Selçuk University, Konya, Turkey

**Keywords:** SARS-CoV-2, COVID-19, regulatory B cells, regulatory T cells

## Abstract

**Background/aim:**

Coronavirus disease 2019 (COVID-19) has a wide clinical spectrum from asymptomatic to mild, moderate, and severe cases. There are still many unknowns about the role of immunoregulatory mechanisms in COVID-19. We aimed to study regulatory T cells (Tregs) and B cell subsets and evaluate their correlations with severity of COVID-19.

**Materials and methods:**

In total, 50 patients with COVID-19 confirmed by PCR (mean age = 49.9 ± 12.8 years) and 40 healthy control (mean age = 47.9 ± 14.7 years) were included in this study. The patients were classified as 14 mild (median age = 35.5 [24–73] years), 22 moderate (median age = 51.5 [28–67] years) and 14 severe (median age = 55.5 [42–67] years). Within 24 h of admission, flow cytometry was used to assess the lymphocyte subsets, Tregs and Bregs without receiving any relevant medication.

**Results:**

In all patients with COVID-19, the proportion of CD3^+^CD8^+^ T cells was reduced (*p* = 0.004) and the CD8^+^ Tregs were increased compared with control (*p* = 0.001). While the levels of regulatory B cells, plasmablasts, and mature naive B cells were found to be significantly high, primarily memory B-cell levels were low in all patients compared with controls (*p* < 0.05). Total CD3^+^ T cells were negatively correlated with the length of stay in the hospital (*r* = −0.286, *p* = 0.044).

**Conclusion:**

The changes in T and B cell subsets may show the dysregulation in the immunity of patients with COVID-19. In this context, the association between CD8^+^ Tregs and COVID-19 severity may help clinicians to predict severe and fatal COVID-19 in hospitalized patients.

## 1. Introduction

The World Health Organization classified the coronavirus disease 2019 (COVID-19) as an outbreak on January 30, 2020 and as a pandemic on March 11, 2020 due to the spread of COVID-19 cases to 220 countries outside of China—the country where the outbreak began [1]. In our country, the first case with COVID-19 was reported on March 11, 2020 after neighboring countries in Europe and Iran [2].

The coronaviruses (CoV) are a large family of viruses that are common worldwide, ranging in severity from the common cold, which can cause self-limiting mild infection manifestations, to more serious infections, such as Middle East respiratory syndrome (MERS) and severe acute respiratory syndrome (SARS) [3]. The clinical findings of the SARS-CoV-2 infection are in the broad spectrum, starting from mild forms, such as asymptomatic disease and mild upper respiratory tract infection, accompanied by respiratory failure and severe viral pneumonia resulting in death [4,5]. It is known that age, sex, and the presence of chronic diseases (such as cardiovascular disease, diabetes, chronic obstructive pulmonary disease, etc.) affect the course of the disease in patients infected with SARS-CoV-2 [6,7].

CD3^+^ T cells, CD3^+^CD4^+^ T cells, and CD3^+^CD8^+^ T cells play a central role in antiviral responses. CD3^+^CD4^+^ T cells promote the production of virus-specific antibodies by activating T-dependent CD19^+^ B cells. CD3^+^CD8^+^ T cells are cytotoxic and can kill viral infected cells. Changes in T lymphocyte subsets of the patients with COVID-19 reported in especially severe cases [7,8]. Lymphopenia commonly correlates with the severity of the disease, especially within the CD8 subset [9]. Predictive immunological data about severe COVID-19 infection is needed to triage these patients. Understanding the disease’s immunological basics may help prevent the progression to severe disease, including cytokine storm and acute respiratory distress syndrome.

During viral infections, host factors trigger an immune response against the virus. However, it should be noted that an uncontrolled immune response may lead to damage of the respiratory tract and other tissues in SARS-CoV infections [8]. Tregs have been shown to limit the extent of virus-induced immunopathology in several virus infections [10,11]. Previous studies have demonstrated that Bregs contribute to immune dysfunction associated with HIV infection via T-cell impairment [12–14]. The effects of B-cell-mediated humoral immunity on the clearance of viral infection are limited, and the role of Bregs is still unclear [15]. In this study, we focused on regulatory T cells (Tregs) and regulatory B cells (Bregs) in patients with COVID-19, and we evaluate their relationship with the clinical findings.

## 2. Materials and methods

### 2.1. Study design and participants

Symptomatic patients with positive SARS-CoV-2 PCR admitted to the Department of Infectious and Chest Diseases from May to June 2020 were recruited in this prospective, single-centered, observational, case-controlled study. Written informed consent was obtained from all patients. Samples were obtained at the time of first admission and viral phase before starting treatment. Peripheral venous blood samples were storage at room temperature before staining. Lymphocyte subsets, Tregs and Bregs, were analyzed by flow cytometry within 24 h. The patients’ demographic data, clinical history, laboratory, radiological findings, and their treatments were obtained from medical records upon admission.

Patients who were under the influence of alcohol or drugs that could cause lymphopenia during treatment, as well as those with chronic renal disease, Cushing syndrome, and/or active cancer, were excluded. The control group consisted of an age and sex-matched healthy adults without the disease. Individuals in the control group with any of the following chronic diseases were also excluded from the study: primary immunodeficiency, chronic kidney or liver disease, active infection, or malignancy. None of them was vaccinated for COVID-19 in the time of study.

COVID-19 patients were divided into three subgroups: mild, moderate, and severe. Those who had mild symptoms or radiologically absent pneumonic findings, were considered as “mild”. Those with fever or respiratory symptoms and radiological signs of pneumonia were considered to be “moderate”. Patients with the following criteria were considered as “severe”: 1) respiratory rate of ≥ 30 breaths/min and dyspnea; 2) SpO_2_ ≤ 93% at rest; 3) PaO_2_/iO_2_ ≤ 300 mmHg; 4) bilateral multilobar, ground glass, and infiltration in radiological imaging (thorax computed tomography); and 5) respiratory failure requiring noninvasive mechanical ventilation [16].

### 2.2. Flow cytometry analysis

**Figure 1:**
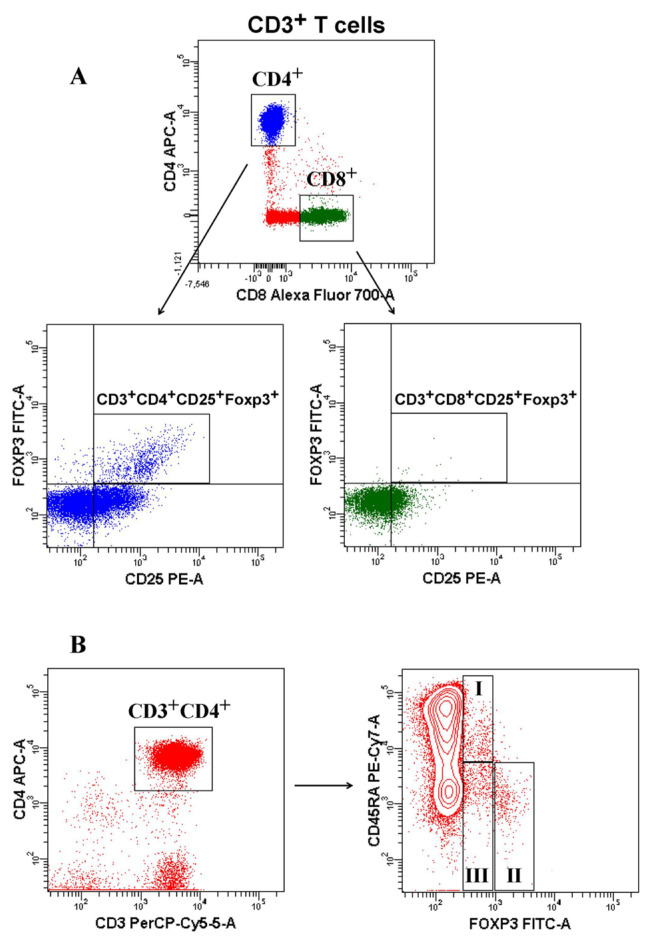
Flow cytometry gating strategy for identification of Treg cells. **A**. CD4^+^ Treg (CD3^+^CD4^+^CD25^+^Foxp3^+^) cells and CD8+ Treg (CD3^+^CD8^+^CD25^+^Foxp3^+^) cells were determined based on the expression of Foxp3^+^ and CD25^+^ in peripheral blood. **B**. Three subsets of CD4^+^ T cells are defined by the expression of CD45RA and Foxp3. I: Resting Treg=rTreg (CD3^+^CD4^+^CD45RA^+^Foxp3^low^), II: Activated Treg=aTreg (CD3^+^CD4^+^CD45RA^-^Foxp3^high^), III: Nonsuppressive Treg=non-Treg (CD3^+^CD4^+^CD45RA^-^Foxp3^low^).

For T-cell subsets and Treg cells, peripheral blood mononuclear cells (PBMCs) were isolated from 2 mL of blood collected in vacutainer glass tubes containing ethylenediaminetetraacetic acid (EDTA). PBMCs were isolated using lymphopure (Biolegend, San Diego, CA). PBMCs were thawed and stained with CD3 (Peridinin chlorophyll protein-Cyanine5.5 (PerCP-Cy5.5), Biolegend, San Diego, CA), CD4 (Allophycocyanin (APC), Becton Dickinson (BD) Bioscience, CA, USA), CD8 (Alexa flour 700, BD Bioscience, CA, USA), CD25 (Phycoerythrin (PE), BD Bioscience, CA, USA), and CD45RA (PE-Cy7, Biolegend, San Diego, CA) extracellularly. Cells were fixed using Foxp3 fixation buffer, stained in permeabilization buffer (BD Bioscience, CA, USA), and labeled with Foxp3 (Fluorescein-5-isothiocyanate (FITC), BD Bioscience, CA, USA) intracellularly. The proportions of CD3^+^, CD3^+^CD4^+^, CD3^+^CD8^+^, CD3^+^CD4^+^CD25^+^Foxp3^+^ (CD4^+^ Tregs), and CD3^+^CD8^+^CD25^+^Foxp3^+^ (CD8^+^ Tregs) were studied in patients and controls (Figure 1A). Also, human Tregs can be divided into three distinct subsets based on Foxp3 level and CD45RA expression [17]: CD3^+^CD4^+^CD45RA^+^Foxp3^low^ (resting Tregs: rTregs), CD3^+^CD4^+^CD45RA^-^Foxp3^high^ (activated Tregs: aTregs), and CD3^+^CD4^+^CD45RA^−^Foxp3^low^ (nonsuppressive Tregs) (Figure 1B).

**Figure 2:**
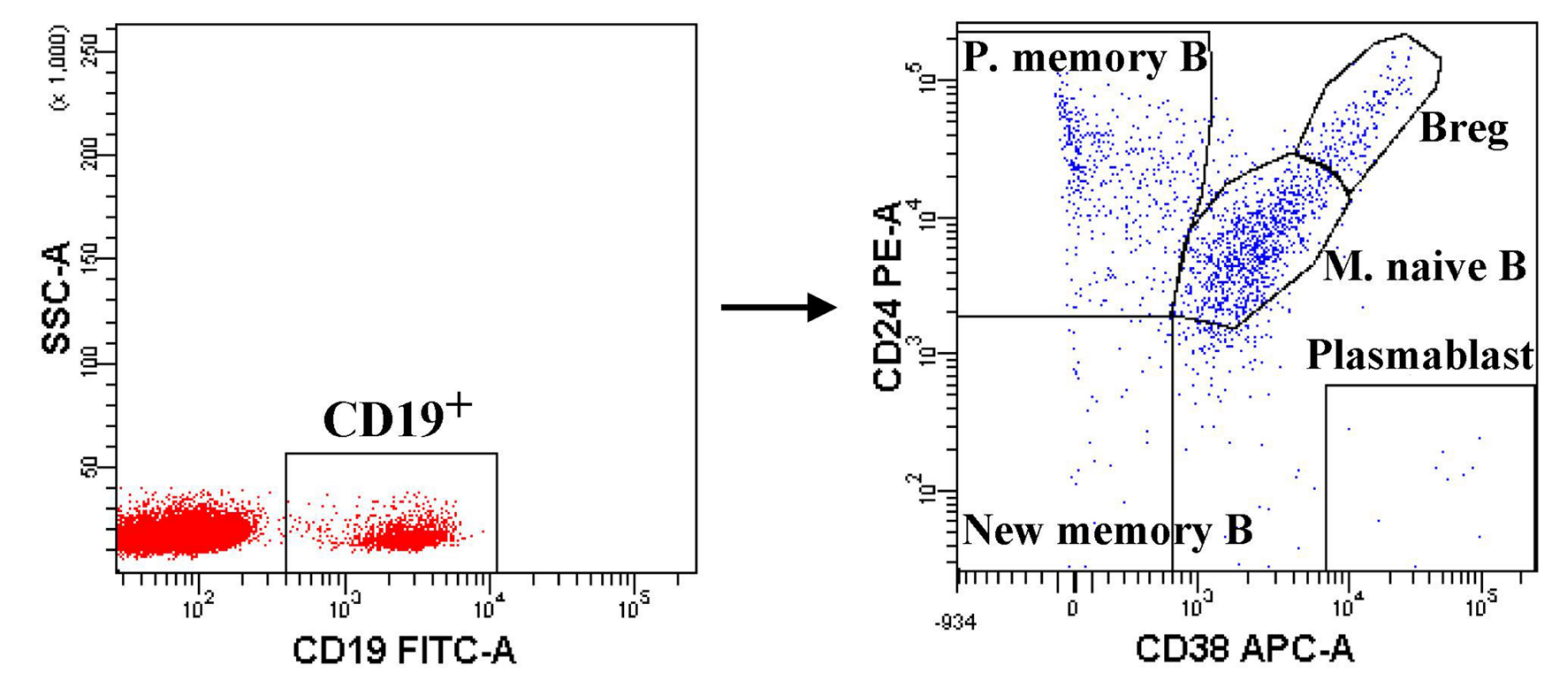
Flow cytometry gating strategy for identification of B cell subsets. The expression of CD24 and CD38 defines five subsets of CD19+ B cells. B regulatory cell (Breg): CD19^+^CD38^high^CD24^high^, Plazmablast: CD19^+^CD38^high^CD24^-^, Mature Naïve B cell: CD19^+^CD38^int^CD24^int^, Primarily Memory B cell: CD19^+^CD38^-^CD24^+^, New memory B cell: CD19^+^CD38^-^CD24^-^.

For the B-cell subsets, peripheral blood samples were collected into vacutainer glass tubes containing EDTA. Blood (100 μL) was transferred into a 12 × 75 mm test tube, and cells were stained with combinations of CD19 (FITC), CD24 (PE), and CD38 (APC) mAbs (all antibodies were obtained from Biolegend, San Diego, CA). The samples were incubated in the dark at room temperature for 20 min. The whole blood lysis method was used. Human B-cell subsets can be divided into five distinct subsets based on CD24 and CD38 expression [18]: CD19^+^CD38^high^CD24^high^ (Bregs), CD19^+^CD38^high^CD24^−^ (plasmablasts), CD19^+^CD38^int^CD24^int^ (mature naïve B cells, int: intermediate), CD19^+^CD38^−^CD24^+^ (primarily memory B cells), and CD19^+^CD38^−^CD24^−^ (new memory B cells) (Figure 2).

Samples were analyzed by dot plots using the Fluorescence-Activated Cell Sorting (FACS) Diva software package (BD Biosciences, CA, USA) with BD FACS ARIA III (BD Biosciences, CA, USA).

### 2.3. Statistical analysis

All analysis was performed using a statistical software package (SPSS for Windows, version 21.0, IBM Corporation, Armonk, NY, USA). Numerical data were expressed as mean ± standard deviation (SD) or median (range: minimum–maximum), as appropriate. Categorical variables were described as count (*n*) and percentages (%). To assess the normality of the data, Shapiro–Wilk’s normality test and Q-Q plots were used. And also, Levene’s test were used to check the homogeneity of the variances.

A Welch *F* test was run to compare the patient ages of the study groups. In addition to, Yates continuity correction chi-square and Fisher’s exact test were conducted to examine the sex and comorbidity distribution between the study groups. Also, we performed the Welch *F* test and Kruskal–Wallis test to compare whether there was a statistically significant difference between the study groups regarding to laboratory and flow cytometry findings. When the variables found to be significant with Welch *F* and Kruskal–Wallis test, we carried out multiple comparisons. The Games-Howell test and Dunn test with Bonferroni correction were applied for pairwise comparisons. Due to the albumin and total bilirubin values were observed only moderate and severe patient groups, we compared these values independent samples t-test. A independent samples *t*-test and Mann–Whitney *U* test were conducted to examine the difference between patients and healthy controls according to flow cytometry findings.

Correlations were analyzed with the Spearman test. Statistical significance was considered at *p*-values less than 0.05.

An a priori power analysis was conducted using “pwr” packages in *R* 3.6.0 (www.r-project.org) to test the difference between two independent groups (COVID patients and controls) using a two-tailed test, a large effect size (*d* =0.80), and an alpha of 0.05. Result showed that a total sample of 34 participants with two equal sized groups of *n* = 68 was required to achieve a power of 0.90. Our study included 50 patients and 40 controls.

## 3. Results

### 3.1. Demographic characteristics

In this study, 50 patients confirmed to have COVID-19 by PCR testing and 40 healthy controls were included. The patient group consisted of 27 females and 23 males. Their ages ranged from 24 to 73 years, with a mean age of 49.9 ± 12.8 years; this age did not differ significantly from the age of the control group (mean age 47.9 ± 14.7 years, range 25 to 82 years) (*p* = 0.482). Also, the sex distribution of the study groups was similar (15 females and 25 males) (*p* = 0.178).

The COVID-19 patients were divided into three subgroups: mild, moderate, and severe. There were 14 mild cases with a median age of 35.5 years (range, 24 to 73 years), 22 moderate cases with a median age of 51.5 years (range, 28 to 67 years), and 14 severe cases with a median age of 55.5 years (range, 42 to 67 years). There was no significant difference in age among groups of patients with COVID-19 (*p* = 0.085) (Table 1).

### 3.2. Clinical and laboratory findings

The most common symptoms were cough (66.0%), fever (36.0%), shortness of breath (34.0%), and fatigue (26.0%). Chills and shivering, sore throat, loss of taste and smell were recorded as the other symptoms. Of the 50 COVID-19 patients, 11 (22.0%) had diabetes, 9 (18.0%) had hypertension, and 4 (8.0%) had asthma. Although the number of patients with hypertension and diabetes in the severe group was higher than in the mild and moderate groups, the difference was not statistically significant (Table 1).

The level of oxygen saturation was below 92.0% in the severe group, and ARDS developed in 7 (50.0%) of them; 5 of these 7 patients received mechanical ventilation treatment in the intensive care unit. The other seven patients improved with high-flow oxygen therapy. In addition to other treatments, six patients followed in the intensive care unit received anticytokine therapy (*n* = 3) and convalescent plasma therapy (*n* = 3). Three patients treated with invasive mechanical ventilation died of respiratory failure on the 13th, 16th, and 18th days of follow-up; two of these patients had received tocilizumab treatment.

There was a significant difference between study groups according to neutrophil, lymphocyte, NLR, prothrombin time, prothrombin-INR, D-dimer, fibrinogen, AST, creatinine phosphokinase, LDH, CRP, ferritin, procalcitonin, troponin I (Table 2). The multiple comparison tests were indicted that the neutrophile counts was higher in severe groups compared to the mild group. The levels of D-dimer, CRP, ferritin, lactate dehydrogenase, troponin I, AST, procalcitonin, and prothrombin time were markedly higher in the severe cases than in the moderate and mild cases. The creatinine phosphokinase, prothrombin-INR and NLR levels of the severe cases were higher than in the moderate cases. The fibrinogen levels of the mild cases were lower than the moderate and severe cases (Table 2).

### 3.3. Radiographic characteristics

Of the mild patients, 14 had no lesions compatible with pneumonia as determined by chest tomography. Unilateral/bilateral pneumonia was detected in 22 patients with COVID-19 of moderate severity. Of the severe cases, 14 had bilateral diffuse ground-glass/consolidation. Typical lesions of the moderate cases were unilateral/bilateral involvement in the form of subpleural ground glass. In severe cases, bilateral multilobar, subpleural, and multifocal localized areas of the ground glass accompanied by interlobular septal thickening showed progression to diffuse ground glass and consolidation areas in both lungs in a short time.

### 3.4. T-cell subsets and regulatory T cells

Lymphopenia (≤1500/mm3) was found in 34/50 (68.0%) patients. According to subgroups, lymphopenia was detected in 71.4% of mild cases (10/14), 50.0% of moderate cases (11/22), and 92.9% of severe cases (13/14). The median value of lymphocyte count in severe cases was lower than moderate cases (1.0 ± 0.3 vs. 1.5 ± 0.6, Games-Howell *p* = 0.004) (Table 2).

**Figure 3:**
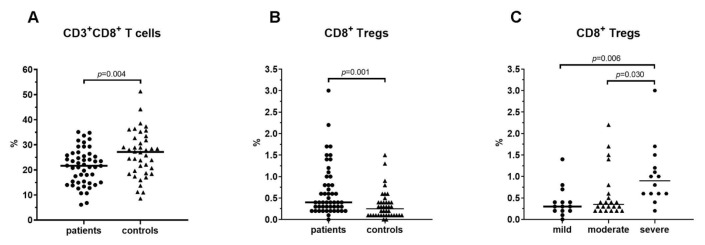
**A**. Percentages of CD8^+^ T cells and **B**. CD8^+^ Tregs in all patients with COVID-19 and healthy controls. **C**. Percentages of CD8^+^ Tregs in the mild, moderate, and severe group in patients with COVID-19.

The percentage of CD3^+^CD8^+^ T cells in all patients (21.3 ± 7.2) was lower than controls (26.3 ± 9.2). This difference was statistically significant (Student’s *t p* = 0.004) (Figure 3A). However, there was no statistically significant difference among the groups with COVID-19 in the percentage of total CD3^+^CD8^+^ T cells (*p* = 0.426)_._ No statistically significant difference was found between all patients and controls regarding in CD4/CD8 ratio and the percentage of total CD3^+^ T cells and CD3^+^CD4^+^ T cells. There was no statistically significant difference among the groups with COVID-19 in the percentage of total CD3^+^ T cells, CD3^+^CD4^+^ T cells and CD4/CD8 ratio (all *p* > 0.05) (Table 3)_._

We illustrated naturally occurring Treg cells (the frequency of CD25^+^Foxp3^+^ Tregs in the total CD3^+^CD4^+^ T cells) in COVID-19 patients and controls in Figure 1A. No statistically significant difference was found between all patients and controls regarding the frequency of Tregs (Mann–Whitney *U p* = 0.202)_._

To determine the frequency of Treg subsets, we could separate three subpopulations’ natural Tregs based on CD45RA and Foxp3 expression in the total CD3^+^CD4^+^ T cells: CD3^+^CD4^+^CD45RA^+^Foxp3^low^ (resting Tregs: rTregs), CD3^+^CD4^+^CD45RA^-^Foxp3^high^ (Activated Tregs: aTregs), and CD3^+^CD4^+^CD45RA^-^Foxp3^low^ (nonsuppressive Tregs) (Figure 1B). The median values of percentage of nonsuppressive Tregs in controls was lower than all patients (3.6 [range, 1.1–11.2] vs. 5 [range, 2.3–13.6], Mann–Whitney *U p* = 0.020). There was no statistically significant difference among the groups with COVID-19 in other subsets of CD4^+^ Tregs (all *p* > 0.05) (Table 3)_._

The median value of percentage of CD8^+^ Tregs (CD3^+^CD8^+^CD25^+^Foxp3^+^) among all patients with COVID-19 were higher than controls (0.4 [range, 0–4.2] vs. 0.3 [range, 0–1.5], Mann–Whitney *U p* = 0.001) (Figure 3B). The percentage of CD8^+^ Tregs of the severe cases (0.9 [range, 0.2–4.2]) was higher than of the mild (0.3 [range, 0–1.4], Bonferroni-Dunn test *p* = 0.006) and moderate cases (0.4 [range, 0.2–2.2], Bonferroni-Dunn test *p* = 0.030) (Figure 3C) (Table 3)_._

### 3.5. B-cell subsets

**Figure 4:**
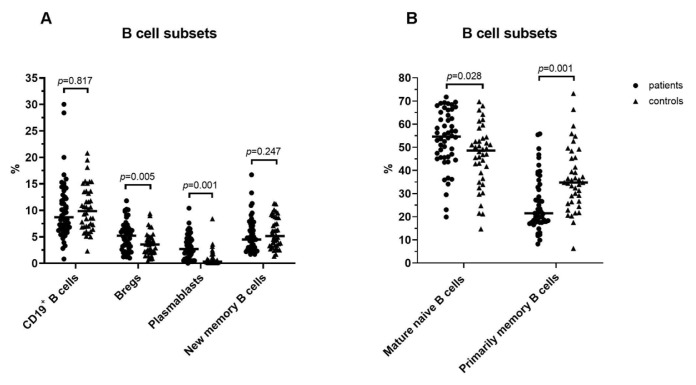
**A**. Percentages of CD19^+^ B cells, Bregs, plazmablasts, and new memory B cells **B**. Mature naïve B cells and primarily memory B cells in all patients with COVID-19 and healthy controls.

Five distinct B-cell populations were determined in the peripheral blood lymphocytes: CD19^+^CD38^high^CD24^high^ (Bregs), CD19^+^CD38^high^CD24^−^ (plasmablasts), CD19^+^CD38^int^CD24^int^ (mature naïve B cells), CD19^+^CD38^-^CD24^+^ (primarily memory B cells), and CD19^+^CD38^−^CD24^−^ (new memory B cells) (Figure 2). The percentage of Bregs (3.6 ± 2.2 vs. 5.2 ± 2.8, Student’s *t p* = 0.005) plasmablasts (0.3 [range, 0.1–8.4] vs. 2.7 [range, 0–10.4], Mann–Whitney *U p* = 0.001) and mature naive B cells (48.6 [range, 14.7–82.8] vs. 54.6 [range, 19.9–71.7], Mann–Whitney *U p* = 0.028) were found to be increased in all patients compared with controls (Figures 4A and 4B). The percentage of primarily memory B cells were found to be decreased in all patients compared with controls (21.5.6 [range, 8.2–55.8] vs. 34.8 [range, 6.3–73.2], Mann–Whitney *U p* = 0.001) (Figure 4B). No statistically significant difference was found between all patients and controls regarding the frequency of new memory B cells (Mann–Whitney *U p* = 0.247) (Figure 4A). In the analysis of subgroups, there was no statistically significant difference among the groups with COVID-19 in all B-cell subsets (all *p* > 0.05) (Table 3)_._

### 3.6. Correlation of the T-cell subsets, Tregs, and Bregs between inflammatory markers

Neutrophil counts were positively correlated with CD4^+^ Tregs (Pearson’s *r* = 0.289, *p* = 0.042), CD8^+^ Tregs (Spearman’s *rho* = 0.436, *p* = 0.002), nonsuppressive Tregs (Spearman’s *rho* = 0.341, *p* = 0.015), CD19^+^ B cells (Pearson’s *r* = 0.492, *p* < 0.001), primarily memory B cells (Spearman’s *rho* = 0.304, *p* = 0.032), and were negatively correlated with Bregs (Pearson’s *r* = −0.475, *p* < 0.001).

Lymphocyte counts were positively correlated with CD3^+^CD8^+^ T cells (Pearson’s *r* = 0.355, *p* = 0.011) and were negatively correlated with CD4/CD8 ratio (Spearman’s *rho* = −0.285, *p* = 0.044).

NLR values were positively correlated with CD8^+^ Tregs (Spearman’s *rho* = 0.367, *p* = 0.009) and were negatively correlated with Total CD3^+^ T cells (Spearman’s *rho* = −0.302, *p* = 0.032), CD3^+^CD8^+^ T cells (Spearman’s *rho* = −0.285, *p* = 0.045), Bregs (Spearman’s *rho* = −0.357, *p* = 0.011) and plasmablasts (Spearman’s *rho* = −0.318, *p* = 0.024).

D-dimer values were positively correlated with CD4^+^ Tregs (Pearson’s *r* = 0.330, *p* = 0.019), CD8^+^ Tregs (Spearman’s *rho* = 0.549, *p* < 0.001) and nonsuppressive Tregs (Spearman’s *rho* = 0.453, *p* = 0.001).

Fibrinogen values were positively correlated with nonsuppressive Tregs (Spearman’s *rho* = 0.508, *p* = 0.001) and were negatively correlated with plasmablasts (Pearson’s *r* = −0.325, *p* = 0.049).

CRP values were positively correlated with CD8^+^ Tregs (Spearman’s *rho* = 0.348, *p* = 0.013) and nonsuppressive Tregs (Spearman’s *rho* = 0.366, *p* = 0.009) and were negatively correlated with Bregs (Spearman’s *rho* = −0.434, *p* = 0.002).

Ferritin values were positively correlated with CD8^+^ Tregs (Spearman’s *rho* = 0.290, *p* = 0.041) and primarily memory B cells (Spearman’s *rho* = 0.313, *p* = 0.027), and were negatively correlated with CD3^+^CD4^+^ T cells (Spearman’s *rho* = −0.295, *p* = 0.038), CD4/CD8 ratio (Spearman’s *rho* = −0.330, *p* = 0.019) and Bregs (Spearman’s *rho* = −0.394, *p* = 0.005).

Serum albumin levels were positively correlated with Bregs (Pearson’s *r* = 0.396, *p* = 0.019).

Serum procalcitonin levels were positively correlated with nonsuppressive Tregs (Spearman’s *rho* = 0.326, *p* = 0.021) and were negatively correlated with Bregs (Spearman’s *rho* = −0.394, *p* = 0.005) and plasmablasts (Spearman’s *rho* = −0.323, *p* = 0.022).

Total CD3^+^ T cells were negatively correlated with length of stay in the hospital (*r* = −0.286, *p* = 0.044) and age (*r* = −0.235, *p* = 0.026). Bregs were negatively correlated with age (*r* = −0.293, *p* = 0.005). We analyzed CD4+ Tregs, CD8+ Tregs, and Bregs with each other about correlation and found no correlation of these cell subtypes.

## 4. Discussion

The molecular mechanisms of Foxp3 expression and the antigen-specific response of CD4^+^ and CD8^+^ Treg cells in COVID-19 remain unclear. The host response to viruses compromises multiple cell types that have a regulatory function. To the best of our knowledge, this is the first study in which all regulatory cells (CD4^+^ Tregs, CD8^+^ Tregs, and Bregs) were evaluated simultaneously in COVID-19 patients. We showed that CD8^+^ Treg cells were increased in all cases, and it was higher in severe cases than the moderate and mild cases. CD8^+^ Tregs have been shown that immunosupressive effects by various mechanisms including direct lysis of target cells and secretion of immune suppressive cytokines [19]. In contrast to nonspecific CD4^+^ Tregs, CD8^+^ Tregs are highly suppressive and described to target only activated T cells for regulation [20,21]. Thus, these results suggested that complementary regulatory mechanisms in the immune system may be affected during SARS-CoV-2 infections.

In the literature, Tregs have been described to limit immune-mediated inflammation and the extent of tissue damage during virus infection [22]. Chen et al. [9] found that the proportion of CD45RA+ Tregs was significantly lower in patients with severe cases than in those with moderate cases (0.5% vs. 1.1%). Another study reported that Treg levels in COVID-19 patients were lower in severe cases [23]. Vick et al. [24] reported that SARS-CoV-2 caused a disruption in the transport of Tregs from the circulation to the respiratory tract and also led to the accumulation of Tregs in the circulation and lung damage. However, opposite findings have also been reported that Treg levels were increased in COVID-19 patients [25,26]. We also observed that nonsuppressive Tregs increased in patients but no other Tregs subsets. The nonsuppressive Tregs are capable of secreting a series of cytokines including interleukin (IL)-2, interferon-γ, and IL-17, but without inhibitory activity [27]. These results showed that the presence of inflammatory and immunosupressive responses in all COVID patients.

In previous studies, although the proportion of T, B, and NK cells was in the normal range in severe and nonsevere cases, decreased numbers of circulating T-cell subsets as a part of lymphopenia have been reported in COVID-19 [9,23,28,29]. We found that the percentage of CD8^+^ T cells was reduced in all patients with COVID-19. CD8^+^ T cells can kill virus-infected cells, and they play a vital role in clearing SARS-CoVs from infected cells and induce immune injury [8]. Several potential mechanisms have been defined, including immune complexes triggered by viral infection, direct infection, promotion of growth inhibition, and apoptosis of these cells. These findings suggested that homeostasis among the T-cell subsets was disrupted in patients with COVID-19.

We detected that the percentages of regulatory B cells, plasmablasts, and mature naive B cells were increased in patients. In addition, primarily memory B cells were decreased in patients. To our knowledge, this is the first study evaluating regulatory B cells of patients with laboratory-confirmed SARS-CoV-2 infection. The effects of B cells on the clearance of viral infections are limited. Only a few studies have investigated the role of Bregs in infections of HIV and hepatitis B virus (HBV) [12–14]. The role of Bregs in viral infections is primarily through IL-10-mediated immunological effects. A previous study reported the B-cell-mediated regulation of T-cell function in HIV infection via suppressing anti-HIV effector CD8^+^ T cells [14]. Jiao et al. [12] found that Bregs frequency correlated negatively with CD4 count, and they determined that Breg phenotypes in HIV-infected patients were CD19^+^CD24^hi^CD38^hi^. The level of IL10 was also significantly higher in this population than the other B-cell subsets [12]. We also identified Bregs according to the CD19^+^CD24^hi^CD38^hi^ population and demonstrated an increase in this population in patients. These observations may be explained by differences in infected individuals’ immune systems and by the effects of SARS-CoV-2 on B-cell subsets.

Consistent with previous reports [5,6], similar clinical features in patients with COVID-19 were noted in the present study. The average age and the number of comorbidities of patients in the severe group, including hypertension and diabetes, were higher than in the nonsevere groups. Although the female patient ratio was higher (54.0%) and lymphopenia (≤ 1500) was detected in 68.0% of all patients, there was no relationship between these parameters and disease severity.

In conclusion, the results presented here demonstrated CD8^+^ Treg cells and Bregs were altered in COVID-19 patients. A better understanding of the immunoregulatory mechanisms may assist in the elucidation of associated factors in the progression of patients infected with COVID-19.

## Figures and Tables

**Table 1:** Demographical and clinical characteristics of the COVID-19 patients.

	All patients (*n* = 50)	Study groups			*p*-value
		Mild (*n* = 14)	Moderate (*n* = 22)	Severe (*n* = 14)	
**Demographic**					
Age, years	49.9 ± 12.8	35.5 (24–73)	51.5 (28–67)	55.5 (42–67)	0.085^1^
Sex (M), n (%)	23 (46.0%)	7 (50.0%)	8 (36.4%)	8 (57.1%)	0.447^2^
**Comorbidities**, n (%)					
Hypertension	9 (18.0%)	1 (7.1%)	4 (18.2%)	4 (28.6%)	0.480^3^
Diabetes	11 (22.0%)	1 (7.1%)	3 (13.6%)	7 (50.0%)	0.049^3^
Asthma	4 (16.0%)	0 (0.0%)	2 (9.1%)	2 (14.3%)	0.548^3^

Data were presented as mean ± standard deviation or median (min-max), as appropriate.

1 Kruskal–Wallis test.

2 Yates continuity correction chi-square test.

3 Fisher-Freeman-Halton chi-square test.

**Table 2:** Laboratory findings of the COVID-19 patients.

	Normal range	All patients (*n* = 50)	Study groups			*p*-value
			Mild (*n* = 14)	Moderate (*n* = 22)	Severe (*n* = 14)	
**Hematological parameters**						
White blood cell count, ×10^9^/L	3.5–10.5	6.0 ± 2.2	5.2 ± 2.1	6.0 ± 2.2	6.8 ± 2.4	0.179^1^
Neutrophil count, ×10^9^/L	1.7–7	3.6 (0.5–10.9)	3 (0.5–7.5)^a^	3.1 (1.3–11)	4.5 (1.9–9.5)^b^	0.043^2^
Lymphocyte count, ×10^9^/L	0.9–2.9	1.3 ± 0.6	1.4 ± 0.9	1.5 ± 0.6^a^	1.0 ± 0.3^b^	0.005^1^
Neutrophil-to-lymphocyte ratio		2.8 (0.5–17)	1.9 (0.5–12.5)	1.9 (0.6–15.6)^a^	4.1 (1.6–17)^b^	0.015^2^
Platelet count, ×10^9^/L	150–450	193.5 (100–727)	180.5 (126–430)	200.5 (100–727)	189 (119–369)	0.278^2^
**Coagulation parameters**						
Prothrombin time, seconds	10–14	11.2 (10–15)	11 (10.2–13.3)^a^	11 (10–14.4)^a^	12.7 (10.7–15)^b^	0.010^2^
Activated partial thromboplastin time, seconds	21–36	26.6 (22.4–42)	26.1 (22.4–35.4)	25.6 (22.9–33)	27.1 (23.2–42)	0.344^2^
Prothrombin-INR	0.8–1.3	0.99 (0.86–1.5)	1 (0.89–1.17)	0.95 (0.86–1.22)^a^	1.11 (0.97–1.5)^b^	0.003^2^
D-dimer, ng/mL	0–500	371.5 (98–1560)	213 (98–499)^a^	348 (100–1130)^a^	531 (310–1560)^b^	0.001^2^
Fibrinogen, mg/dL	200–400	419.2 ± 172.6	310.8 ± 59.7^a^	404.8 ± 121.4^b^	599.6 ± 217.4^b^	0.002^1^
**Biochemical parameters and acute phase reactants**						
Alanine aminotransferase, U/L	0–50	24.5 (8–192)	18.5 (8–58)	22.5 (9–192)	34.5 (11–64)	0.185^2^
Aspartate aminotransferase,U/L	0–50	28 (13–148)	21.5 (14–38)^a^	26 (13–148)^a^	35 (18–91)^b^	0.014^2^
Albumin, g/dL	3.5–5.2	3.6 ± 0.4	-	3.7 ± 0.4	3.5 ± 0.4	0.229^3^
Total bilirubin, mg/dL	0.22–1.4	0.5 ± 0.2	-	0.4 ± 0.2	0.6 ± 0.2	0.083^3^
Urea, mg/dL	17–43	30.5 (13–120)	25 (14–120)	32 (13–59)	33 (22–98)	0.222^2^
Creatinine, mg/dL	0.67–1.2	0.82 (0.58–4.83)	0.8 (0.6–4.8)	0.8 (0.6–1.2)	0.9 (0.7–1.8)	0.126^2^
Creatinine phosphokinase	34–171	85 (28–584)	92.5 (28–211)	57 (34–370)^a^	154 (48–584)^b^	0.019^2^
Lactate dehydrogenase, U/L	126–222	276.2 ± 114.3	196.1 ± 46.3^a^	256.7 ± 66.3^b^	386.7 ± 138.9^c^	0.001^1^
Ferritin, ng/mL	23.9–336.2	114.2 (2.7–842.2)	74.9 (2.7–408)^a^	99.6 (6.1–584.7)^a^	230.7 (21.2–842.2)^b^	0.010^2^
C-reactive protein, mg/L	0–8	9.86 (1.3–462)	4.9 (1.6–55.5)^a^	8.4 (1.3–156)^a^	119.3 (11.6–462)^b^	0.001^2^
Procalcitonin, ng/mL	0–0.5	0.05 (0.05–1.25)	0.05 (0.05–1.25)^a^	0.05 (0.05–0.20)^a^	0.1 (0.05–0.64)^b^	0.012^2^
Troponin I, ng/L	0–17.5	2.9 (2.2–12)	2.8 (2.3–5.3)^a^	2.4 (2.2–9.5)^a^	5.1 (2.3–12)^b^	0.015^2^

Data were presented as mean ± standard deviation or median (min-max), as appropriate.

Different small superscript in each row denote that statistically significant between groups after pairwise comparisons.

1 Welch *F* test followed by Games-Howell posthoc test

2 Kruskal–Wallis test followed by Dunn posthoc test with Bonferroni correction.

3 Independent samples *t*-test.

**Table 3:** The regulatory T Cells and B Cell subsets of the COVID-19 patients and healthy controls.

	Control (*n* = 50)	All patients (*n* = 50)	*p* value	Study groups			*p*-value
				Mild (*n* = 14)	Moderate (*n* = 22)	Severe (*n* = 14)	
**Lymphocyte gate**							
CD3^+^ Total T (%)	71.4 ± 8.1	68.0 ± 10.3	0.088^1^	73 (47.6–78.6)	70.9 (48–85.3)	64.6 (36.9–81.1)	0.055^3^
CD3^+^CD4^+^Th (%)	40.7 ± 7.9	40.0 ± 8.8	0.726^1^	40.2 ± 8.8	41.3 ± 9.0	37.9 ± 9.0	0.537^4^
CD3^+^CD8^+^Tc (%)	26.3 ± 9.2	21.3 ± 7.2	0.004^1^	22.9 ± 7.2	21.5 ± 7.7	19.4 ± 6.2	0.426^4^
CD4/CD8 ratio	1.6 (0.5–5.4)	1.7 (0.9–8.7)	0.099^2^	1.7 (0.9–4.1)	1.9 (0.9–8.7)	1.8 (1.1–3.9)	0.715^3^
**CD3** **^+^** **CD4** **^+^** **Th gate (%)**							
CD3^+^CD4^+^CD25^+^Foxp3^+^(Tregs)	5.2 (1.6–15)	6.4 (1.3–10.9)	0.202^2^	6.1 ± 2.0	6.6 ± 1.6	6.4 ± 2.5	0.758^4^
**I:** CD3^+^CD4^+^CD45RA^+^Foxp3^low^ (RestingTregs=rTregs)	1.2 ± 0.8	1.3 ± 1.0	0.922^1^	1.7 ± 1.4	1.1 ± 0.8	1.1 ± 0.5	0.290^5^
**II:** CD3^+^CD4^+^CD45RA^-^Foxp3^high^ (ActivatedTregs=aTregs)	1.5 ± 1.7	1.7 ± 1.3	0.134^1^	1.6 (0.5–3.6)	1.8 (0.2–4.7)	1.2 (0.3–8.1)	0.664^3^
**III:** CD3^+^CD4^+^CD45RA^−^Foxp3^low^ (Nonsuppressive Tregs)	3.6 (1.1–11.2)	5 (2.3–13.6)	0.020^2^	3.9 (2.4–6.9)	5.5 (2.6–8.5)	5.2 (2.3–13.6)	0.173^3^
**CD3** **^+^** **CD8** **^+^** **Tc gate (%)**							
CD3^+^CD8^+^CD25^+^Foxp3^+^	0.3 (0–1.5)	0.4 (0–4.2)	0.001^2^	0.3 (0–1.4)^a^	0.4 (0.2–2.2)^a^	0.9 (0.2–4.2)^b^	0.005^3^
**Lymphocyte gate (%)**							
CD19^+^ B cells	10.3 ± 4.3	10.1 ± 5.6	0.817^1^	7.9 ± 3.2	11.5 ± 7.1	10.0 ± 4.4	0.166^4^
**CD19** **^+^** ** B gate (%)**							
CD19^+^CD38^high^CD24^high^ (Bregs)	3.6 ± 2.2	5.2 ± 2.8	0.005^1^	6.3 ± 2.7	4.8 ± 3.0	4.6 ± 2.2	0.208^4^
CD19^+^CD38^high^CD24^−^ (Plasmablasts)	0.3 (0.1–8.4)	2.7 (0–10.4)	0.001^1^	3.2 ± 2.1	3.2 ± 2.2	2.9 ± 2.6	0.913^4^
CD19^+^CD38^int^CD24^int^ (Mature naive B cells)	48.6 (14.7–82.8)	54.6 (19.9–71.7)	0.028^2^	51.9 ± 11.4	56.1 ± 10.9	50.7 ± 15.5	0.393^4^
CD19^+^CD38^−^CD24^+^ (Primarily memory B cells)	34.8 (6.3–73.2)	21.5 (8.2–55.8)	0.001^2^	21.9 (9.9–55.8)	19.8 (8.2–46.8)	23.6 (12.6–55.4)	0.504^3^
CD19^+^CD38^-^CD24^−^ (New memory B cells)	5.2 (1.3–11.3)	4.5 (1.7–16.7)	0.247^2^	5.1 (2.2–11.3)	3.5 (1.7–9.5)	4.9 (1.7–16.7)	0.283^3^

Data were presented as mean ± standard deviation or median (min-max), as appropriate.

Different small superscript in each row denote that statistically significant between groups after pairwise comparisons.

1 Independent samples *t*-test.

2 Mann–Whitney *U* test.

3 One-way ANOVA followed by Tukey HSD posthoc test.

4 Kruskal–Wallis test followed by Dunn posthoc test with Bonferroni correction.

5 Welch *F* test followed by Games-Howell posthoc test.
